# Supplementary material for: Observation of triplet-assisted long-distance charge-transfer exciton transport in single organic cocrystal
Source: Nat Commun. 2025 Aug 29;16:8081. doi: 10.1038/s41467-025-63388-0 (PMC12397298; doi:10.1038/s41467-025-63388-0)
Supplement: Supplementary file 1 — Supplementary Information [file 41467_2025_63388_MOESM1_ESM.pdf]

Supplementary Information for

Observation of triplet-assisted long-distance charge-transfer exciton transport in single organic cocrystal

Yejun Xiao*[,1], Xianchang Yan[1,2], Rui Cai[3], Xuan Liu[4], Jingwen Bao[1], Min Zhang[1,2], Jing Leng*[,1], Shengye Jin*[,1], and Wenming Tian*[,1]

[1] State Key Laboratory of Chemical Reaction Dynamics, Dalian Institute of Chemical Physics, Chinese Academy of Sciences

Dalian 116023, China

[2] University of Chinese Academy of Sciences

Beijing 100049, China

[3] Instrumental Analysis Center, Dalian University of Technology

Dalian 116024, China

[4] State Key Laboratory of Catalysis, Dalian National Laboratory for Clean Energy, Dalian Institute of Chemical Physics, Chinese Academy of Sciences

Dalian 116023, China

Corresponding Author

yjxiao@dicp.ac.cn

ljjx@dicp.ac.cn

sjin@dicp.ac.cn

tianwm@dicp.ac.cn

Supplementary Note 1

Estimations of exciton diffusion parameters and the resolution of our PL imaging measurements

The exciton diffusion in lateral dimension can be described by a two-dimensional diffusion equation:

$$\frac{\partial n(x,y,t)}{\partial t} = D\left\{\frac{\partial^2 n(x,y,t)}{\partial x^2} + \frac{\partial^2 n(x,y,t)}{\partial y^2}\right\} - k_1 n(x,y,t) \quad (1)$$

where $n(x, y, t)$ is the concentration of excitons at time t and position (x, y); D is the diffusion coefficient, k_1 is the first order recombination coefficient. As the initial exciton population follows a Gaussian-type distribution due to the Gaussian pump beam, a general solution to this diffusion equation is:

$$n(x,y,t) = \frac{1}{4\pi Dt}\exp\left(-\frac{x^2+y^2}{4Dt}\right) \quad (2)$$

In terms of Equation 2 and Equation 1 in the main text, the Gaussian variance of 1D PL profile extracted from PL image (i.e., the cross section of PL image) can be directly related to the diffusion coefficient D as follows:

$$\sigma_{x,t}^2 = \sigma_{x,0}^2 + l_x^2 = \sigma_{x,0}^2 + 2Dt \quad (3)$$

where l_x is the distance of exciton diffusing away (along x-direction) from the initial position at delay time t. Therefore, the diffusion coefficient D can be directly obtained from the linear fitting of Gaussian variances at different times:

$$D = \frac{\sigma_{x,t}^2 - \sigma_{x,0}^2}{2t} = \frac{l_x^2}{2t} \quad (4)$$

and the 2D diffusion distance L_D can be estimated by $L_D = \sqrt{l_x^2 + l_y^2} = 2\sqrt{D\tau}$.

The spatial resolution of this exciton transport imaging method is determined by the signal-to-noise of the setup coming from the uncertainty of the Gaussian profiles measured at different time ($\Delta\sigma_t$ and $\Delta\sigma_0$), rather than the diffraction limit:[1]

$$\Delta L = \sqrt{\frac{\sigma_t^2}{\sigma_t^2 - \sigma_0^2}\Delta\sigma_t^2 + \frac{\sigma_0^2}{\sigma_t^2 - \sigma_0^2}\Delta\sigma_0^2} \quad (5)$$

Therefore, based on the uncertainty of Gaussian fitting, the spatial resolution limit in our experiments is ~58 nm for PL imaging and ~279 nm for delayed PL imaging.

Supplementary Note 2

Charge-transfer exciton kinetic model in T_S-T_C

From the possible photophysical processes in T_S-T_C as illustrated in Fig. 3a, the singlet and triplet exciton populations are coupled and described by the following excited state reactions:

$$^1\text{CT} \overset{k_1}{\rightarrow} {}^3\text{CT} \tag{6}$$

$$^3\text{CT} \overset{k_2}{\rightarrow} {}^1\text{CT} \tag{7}$$

$$^1\text{CT} \overset{k_4}{\rightarrow} \text{T}_1 \tag{8}$$

$$\text{T}_1 \overset{k_5}{\rightarrow} {}^1\text{CT} \tag{9}$$

In addition, recombination process of ^{1}CT, ^{3}CT and T_1 states were also considered and their corresponding rate constants are k_0, k_3 and k_6, respectively. Accordingly, the rate equations of ^{1}CT, ^{3}CT and T_1 populations can be written as:

$$\frac{dn(^1\text{CT})}{dt} = -k_0 n(^1\text{CT}) - k_1 n(^1\text{CT}) - k_4 n(^1\text{CT}) + k_2 n(^3\text{CT}) + k_5 n(\text{T}_1) \tag{10}$$

$$\frac{dn(^3\text{CT})}{dt} = k_1 n(^1\text{CT}) - k_2 n(^3\text{CT}) - k_3 n(^3\text{CT}) \tag{11}$$

$$\frac{dn(\text{T}_1)}{dt} = k_4 n(^1\text{CT}) - k_5 n(\text{T}_1) - k_6 n(\text{T}_1) \tag{12}$$

We first assumed a reported double-channel ISC mechanism where both of T_1 and ^{3}CT are in dynamic equilibrium with ^{1}CT (see Supplementary Fig. 14 for illustration). Using the steady-state approximation method for ^{3}CT and ^{1}T state ($\frac{dn(^3\text{CT})}{dt} \approx 0$ and $\frac{dn(\text{T}_1)}{dt} \approx 0$), following relationships can be obtained on condition that k_1, $k_2 \gg k_3$ and k_4, $k_5 \gg k_6$:

$$n(^3\text{CT}) = \frac{k_1}{k_2} n(^1\text{CT}) \tag{13}$$

$$n(\text{T}_1) = \frac{k_4}{k_5} n(^1\text{CT}) \tag{14}$$

Therefore,

$$\frac{d(n(^1\text{CT}) + n(^3\text{CT}) + n(\text{T}_1))}{dt} = -k_0 n(^1\text{CT}) - k_3 n(^3\text{CT}) - k_6 n(\text{T}_1) \tag{15}$$

Putting Supplementary Equation 13 and 14 into Supplementary Equation 15, the reaction rate can be written as:

$$\frac{dn(^1\mathrm{CT})}{dt} = -\left(\frac{k_0+k_3\frac{k_1}{k_2}+k_6\frac{k_4}{k_5}}{1+\frac{k_1}{k_2}+\frac{k_4}{k_5}}\right) n(^1\mathrm{CT}) \tag{16}$$

It can be seen that the TADF kinetics still exhibits the monoexponential decay property even if there are two ISC pathways.

Further considering the energy order switch of T_1 and ^{1}CT before and after the excitation relaxation, T_1 excitons will actually undergo a favorable RISC process to regenerate ^{1}CT excitons on the same time scale as the ^{1}CT recombination. Therefore, the T_1 contribution can be ignored and Supplementary Equation 16 can be simplified as:

$$\frac{dn(^1\mathrm{CT})}{dt} = -\left(\frac{k_0k_2+k_1k_3}{k_1+k_2}\right) n(^1\mathrm{CT}) \tag{17}$$

$$k_{steady} = k_{DF} = \frac{k_0k_2+k_1k_3}{k_1+k_2} \tag{18}$$

where k_{DF} is the rate constant of the TADF decay.

Estimation of rate constants in T_S-T_C

Under assumption of $k_1 \gg k_2$, Supplementary Equation 18 can be reduced to

$$k_{DF} = k_3 + k_0\frac{k_2}{k_1} \tag{19}$$

The $\frac{k_2}{k_1}$ can be further calculated by:

$$\frac{k_2}{k_1} = \frac{n(^1\mathrm{CT})}{n(^3\mathrm{CT})} = \frac{\emptyset_{DF}k_{DF}}{\emptyset_{PF}k_{PF}} \tag{20}$$

where k_{PF} is the rate constant of prompt fluorescence decay; $\emptyset_{PF}$ and $\emptyset_{DF}$ are the proportion of prompt fluorescence and TADF components respectively. k_{PF}, $\emptyset_{PF}$ and $\emptyset_{DF}$ are given as:

$$k_{PF} = k_0 + k_1 \tag{21}$$

$$\emptyset_{PF} = \frac{k_0^r}{k_0+k_1} \tag{22}$$

$$\emptyset_{DF} = \frac{k_1}{k_0+k_1}\cdot\frac{k_0^rk_2}{k_0k_2+k_1k_3} = \frac{k_0^r}{k_0+k_1}\cdot\frac{k_1k_2}{k_0k_2+k_1k_3} \tag{23}$$

where k_0^r is the radiative recombination rate constant of [1]CT state. Putting Supplementary Equation 22 into Supplementary Equation 23, $\emptyset_{DF}$ can be written as:

$$\emptyset_{DF} = \emptyset_{PF} \cdot \frac{k_1 k_2}{k_0 k_2 + k_1 k_3} \tag{24}$$

From the experimentally obtained values $k_{PF} = 3.44\times10^7$ s^{-1}, $k_{DF} = 1.22\times10^3$ s^{-1}, $\emptyset_{PF} = 0.19$ and $\emptyset_{DF} = 0.81$, $\frac{k_2}{k_1} \approx 1.42\times10^{-4}$ can be calculated. Then, putting these values into Supplementary Equation 19, 21 and 24, $k_0 = 0.01\times10^7$ s^{-1}, $k_1 = 3.43\times10^7$ s^{-1}, $k_2 = 4.88\times10^3$ s^{-1} and $k_3 = 1.21\times10^3$ s^{-1} can be determined. It can be noticed of $k_3 \gg k_0 \frac{k_2}{k_1}$, which confirms the k_3-dominant TADF decay of T_S-T_C.

Estimation of the triplet yield in T_S-T_C

Based on the k_3-dominant TADF decay behavior in T_S-T_C, we can infer that $k_1 k_3 \gg k_0 k_2$, and thus the Supplementary Equation 24 can be simplified as:

$$\emptyset_{DF} = \emptyset_{PF} \cdot \frac{k_1 k_2}{k_0 k_2 + k_1 k_3} \approx \emptyset_{PF} \cdot \frac{k_2}{k_3} \tag{25}$$

Accordingly, the following relation can be obtained:

$$\frac{k_1}{k_0} \gg \frac{k_2}{k_3} \approx \frac{\emptyset_{DF}}{\emptyset_{PF}} \tag{26}$$

The $\frac{\emptyset_{DF}}{\emptyset_{PF}}$ of ~4.3 can be obtained from the ratio of the integral areas as shown in Supplementary Fig. 7b, and then the lower limit of the triplet yield ($\emptyset_T$) in T_S-T_C can be estimated as:

$$\emptyset_T = \frac{k_1}{k_0 + k_1} = \frac{\frac{k_1}{k_0}}{\frac{k_1}{k_0} + 1} > \frac{4.3}{4.3+1} \approx 0.81 \tag{27}$$

Supplementary Note 3

Simulations of 2D ^{3}CT exciton diffusion dynamics in T_S-T_C

The in-plane exciton diffusion in T_S-T_C shown in Fig. 3d can be described by a 2D diffusion model (see Supplementary Equation 1), and the boundary condition for the diffusion is defined as:

$$\frac{\partial n^{+}(L_x,y,t)}{\partial x} = \frac{\partial n^{-}(0,y,t)}{\partial x} = \frac{\partial n^{+}(x,L_y,t)}{\partial y} = \frac{\partial n^{-}(x,0,t)}{\partial y} = 0 \tag{28}$$

The + and – indicate the forward and backward first-order differential. The initial (t = 0) distribution of excitons at the excitation site can be further described by the Gaussian function:

$$n(x,y,0)_{exc.} = n(x_0,y_0,0)\exp\left(-2\frac{(x-x_0)^2+(y-y_0)^2}{r^2}\right) \tag{29}$$

$$\int n(x,y,0)_{exc.} dxdy = n_0 \tag{30}$$

where $n(x_0,y_0,0)$ is the exciton density at the center of the distribution; r is the distribution radius; n_0 is the initial exciton density. The fitting of the ^{3}CT exciton diffusion dynamics is performed by a home-built program. The side length of the T_S-T_C cocrystal was measured to be 16 μm; the excitation site and the distribution radius (~3.5 μm under focused excitation and ~6.3 μm under defocused excitation) were determined from the TADF intensity images as shown in Supplementary Fig. 9b and Fig. 16. From this fitting, we yielded an intrinsic ^{3}CT lifetime of 850 μs and ^{3}CT diffusion coefficient of 3.5×10^{-4} cm^2/s (Fig. 3d). The fitted D value and ^{3}CT lifetime are well consistent with the experimentally obtained ones (3.8×10^{-4} cm^2/s and 826.4 μs, respectively).

Supplementary Note 4

Simulations of 1D ^{1}CT exciton diffusion dynamics in T_S-T_C

The PL kinetics in T_S-T_C shown in Supplementary Fig. 21 can be described by a 1D diffusion model:

$$\frac{\partial n(x,t)}{\partial t} = D\left\{\frac{\partial^2 n(x,t)}{\partial x^2}\right\} - k_1 n(x,t) \tag{31}$$

where $n(x,t)$ is the density of excitons at time *t* and position *x*. The quenching effect on exciton dynamics in T_S-T_C is included by assuming all excitons reaching the T_S-T_C/PEDOT interface are quenched with 100% efficiency in view of the much faster interfacial hole transfer time with respect to the ^{1}CT exciton lifetime. Based on the diffusion equation, the exciton density at delay time *t* and any thickness inside the T_S-T_C cocrystal was simulated by a home-built program, which can be calculated by the following equation:

$$I_{PL}(t) = \int n(x,t)dx \tag{32}$$

The boundary condition for the diffusion is defined as:

$$\frac{\partial n^+(L,t)}{\partial x} = \frac{\partial n^-(0,t)}{\partial x} = 0 \tag{33}$$

where L is the thickness of T_S-T_C cocrystal; the + and – represent the forward and backward first-order differential, indicating the excitons do not diffuse out of the T_S-T_C cocrystal boundary. The initial (t = 0) distribution of excitons in the interior of T_S-T_C cocrystal can be described by:

$$n(z,0) = n_0 \exp(-\varepsilon z) \tag{34}$$

$$n_0 = \alpha\varepsilon J(1 - R_{pump}) \tag{35}$$

where ε is the absorption coefficient of T_S-T_C which is measured to be 800 cm^{-1}; α is the ratio of exciton created per photon absorbed, which is assumed to be 1; R_{pump} is the reflectivity of T_S-T_C cocrystal at normal incidence of the excitation beam.

Supplementary Figures

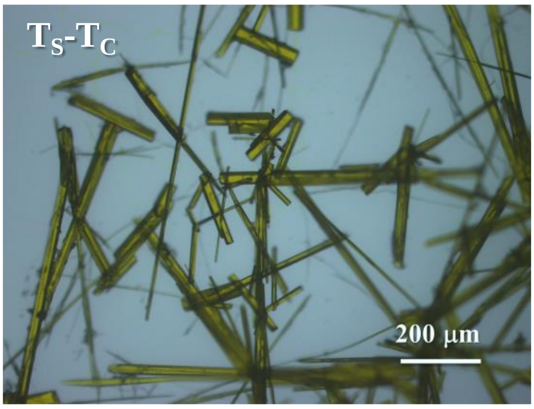

Supplementary Fig. 1 Optical image of T_S-T_C cocrystals.

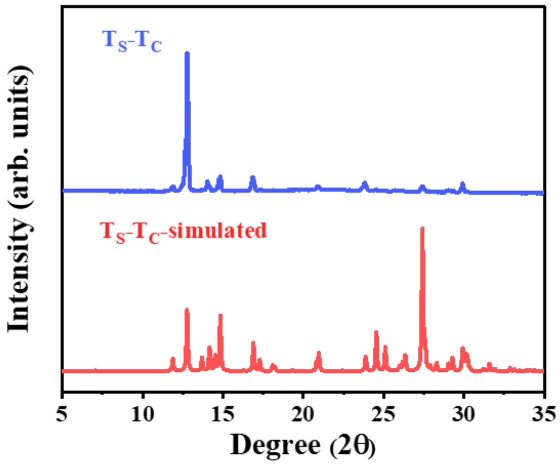

Supplementary Fig. 2 XRD patterns of T_S-T_C and T_S-T_C-simulated.

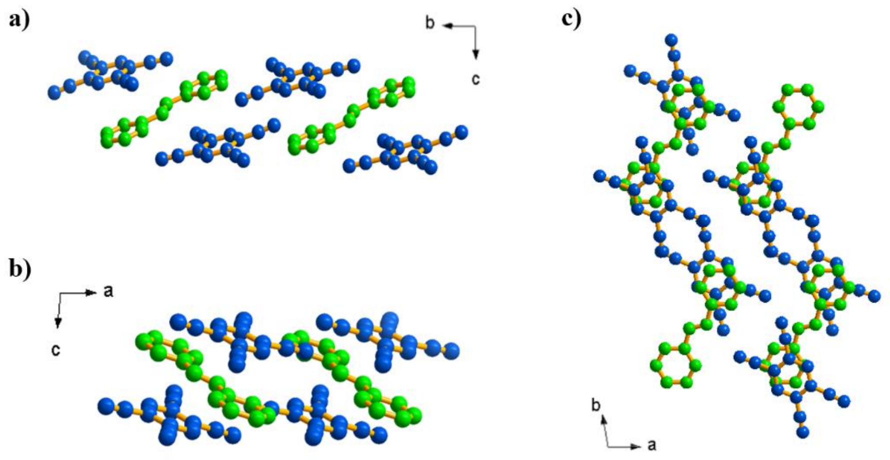

Supplementary Fig. 3 Single crystal structures of T_S-T_C viewing along the (a) *a* axis, (b) *b* axis and (c) *c* axis. Color representation: green, TSB; blue, TCNB.

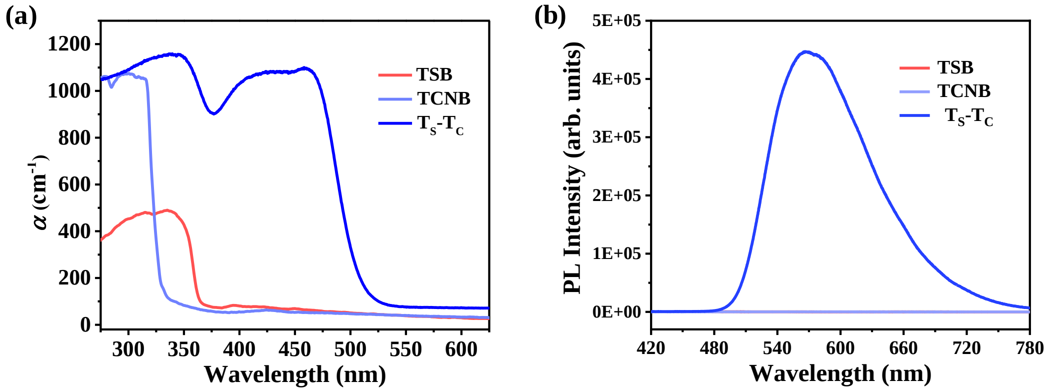

Supplementary Fig. 4 The comparison of absorption and emission properties of TSB, TCNB and T_S-T_C cocrystal: (a) the absorption coefficient (α) and (b) the photoluminescence spectra. The apparent red-shifted absorption and PL emission indicate the CT nature of T_S-T_C cocrystal.

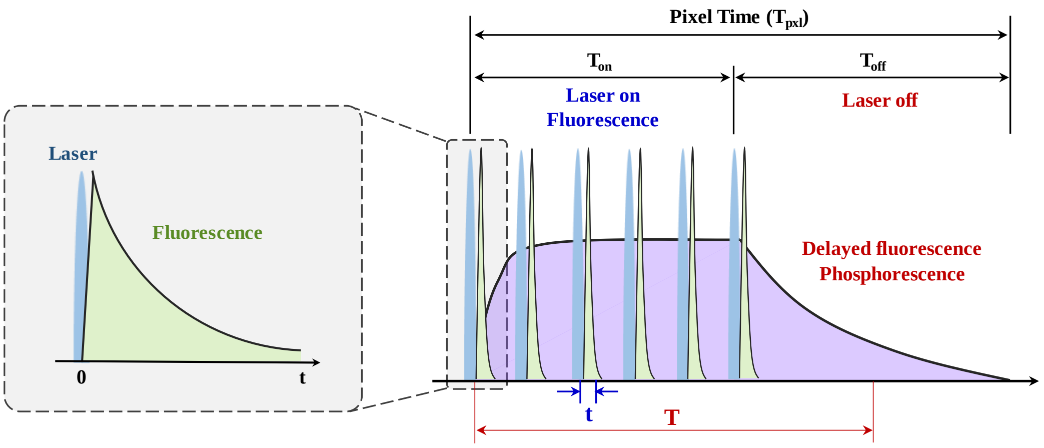

Supplementary Fig. 5 Principle of multipulse-excited time-correlated single photon counting (TCSPC) phosphorescence decay recording (PDR) technique. A high-frequency pulsed laser is on-off modulated synchronously with the pixels. Within the on-time, T_{on}, the laser pulses excite fluorescence, and, pulse by pulse, build up the long-lived PL. The long-lived PL intensity at the end of the laser-on time is far higher than for a single laser pulse. For the rest of the pixel time the laser is turned off. After the last laser pulse, the fluorescence decays quickly, and for the rest of the pixel dwell time, T_{off}, the pure long-lived PL is detected.

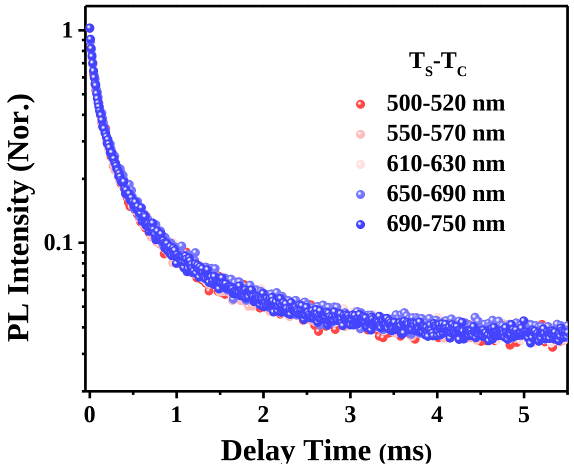

Supplementary Fig. 6 Comparison of ms-scale PL kinetics collected at different wavelength ranges under the 375 nm excitation. The long-lived PL kinetics of T_S-T_C are almost unchanged in regardless of the wavelength range, indicating the sole TADF emission.

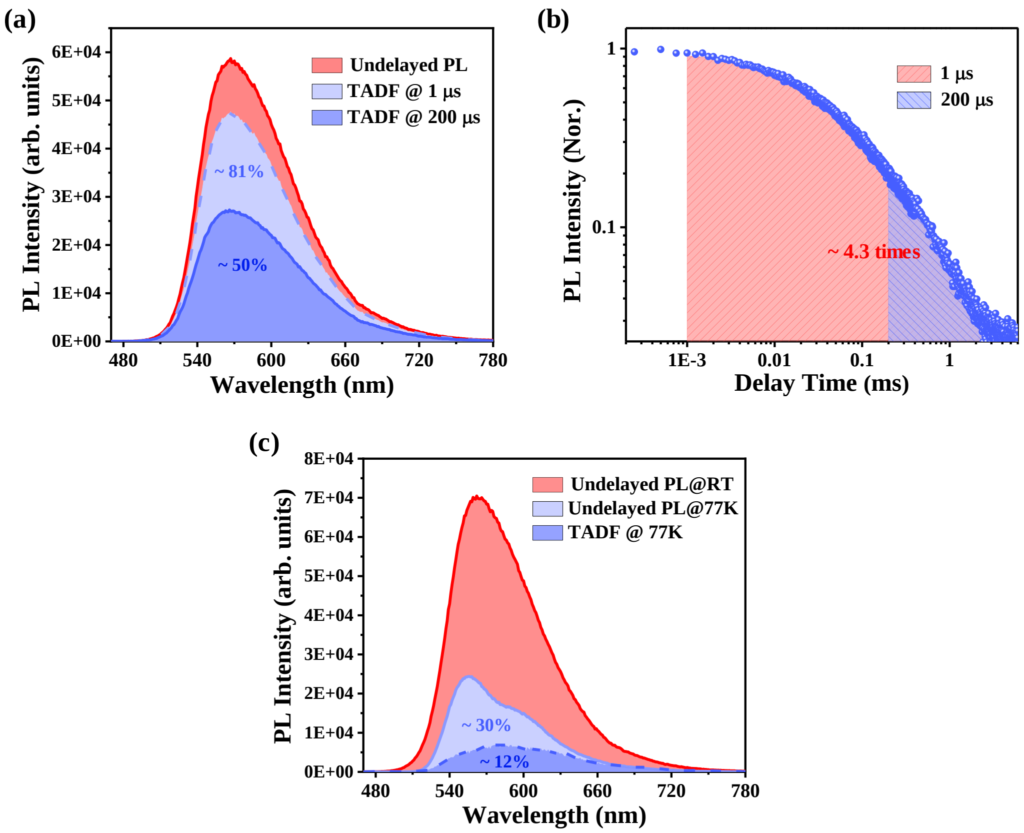

Supplementary Fig. 7 The determination of TADF proportion. (a) Undelayed PL spectra and TADF spectra delayed by 200 μs of T_S-T_C; the dash line is the simulated TADF spectra of T_S-T_C dealyed by 1 μs. (b) Normalized PL decay kinetics of T_S-T_C covering nanosecond to microsecond time window. The PL spectra of T_S-T_C exhibits contributions from both singlet and triplet states, while the TADF spectra only reflects the contribution from the triplet state. The TADF proportion can be estimated to be ~50% at 200 μs delay time based on the ratio of integrated spectral areas of PL and TADF. This result is obviously underestimated; however, the collection of TADF spectra at a shorter delay time is impractical due to the limitation of instrument time resolution. According to the PL kinetics from ns to μs time scale, the weight of triplet state at 1 μs (where fluorescence has been completely decayed) is determined to be ~4.3 times larger than that at 200 μs. Therefore, the TADF proportion at 1 μs can be finally estimated to be ~81%. (c) Undelayed PL spectra collected at room temperature (RT) and 77 K, the dash line is the simulated TADF spectra at 77 K delayed by 1 μs based on the weight

of triplet state estimated from the Supplementary Fig. 7b. Accordingly, the singlet population can be estimated to be ~18%, and an emissive triplet propotion of ~82% can be further obtained which is in consistent with the estimation (~81%) conducted based on Supplementary Fig. 7a.

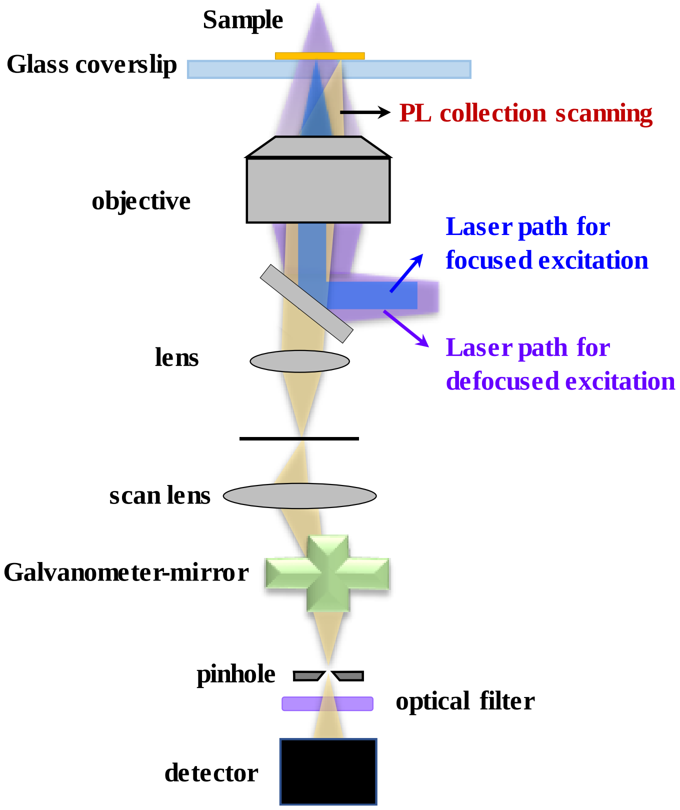

Supplementary Fig. 8 A schematic presentation of the time-resolved and PL-scanned imaging microscopy for PL kinetics imaging measurement. Both of wide-field (defocused) and focused illumination modules are contained. Scanning the PL collection spot on the sample by rotating the galvanometer mirror allows the construction of a PL intensity image.

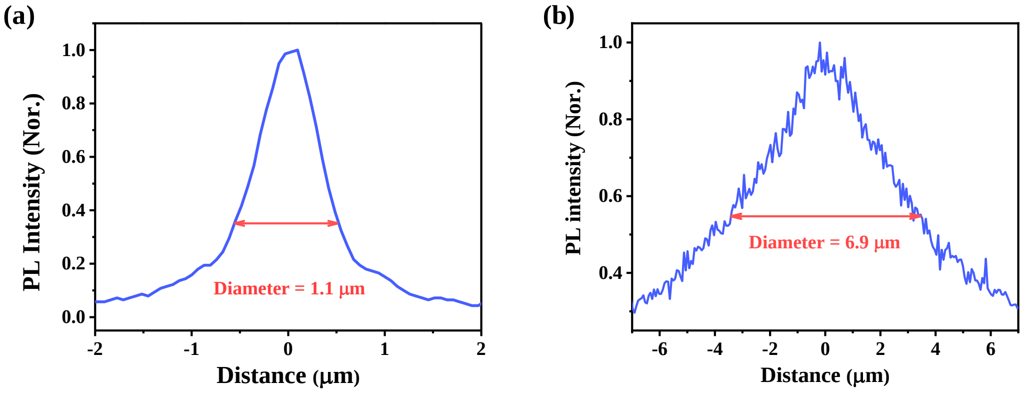

Supplementary Fig. 9 Diameter of (a) the initial PL distribution at t = 0 ns after the focused excitation, estimated to be ~1.1 μm; (b) the initial TADF distribution at t = 17.5 μs after the focused excitation, estimated to be ~6.9 μm.

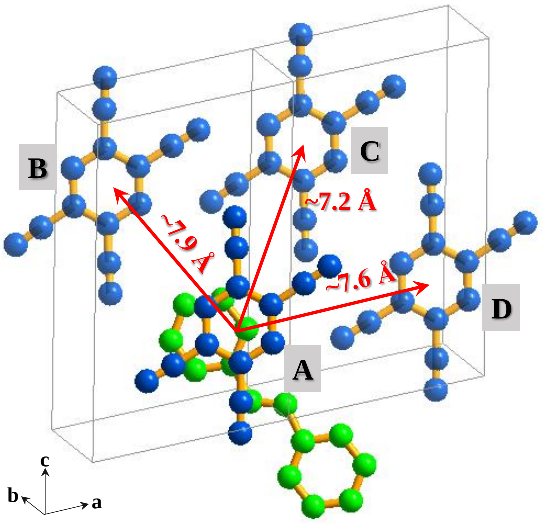

Supplementary Fig. 10 The three potential CT transport directions in T_S-T_C based on its crystal structure. Although the unit cell parameters of T_S-T_C are isotropic (see Supplementary Table 1), the actual distances for CT exciton hopping between adjacent D-A units AB, AC and AD along the directions of $\overrightarrow{ac}$, $\overrightarrow{bc}$ and $\vec{a}$ are measured to be similar, which are ~7.9 Å, 7.6 Å and 7.2 Å, respectively. In view of the distance-dependence feature of Dexter energy transfer, the comparable hopping distances along different directions are expected to result in the isotropic CT diffusion in T_S-T_C. Note: the center-to-center distances between pairs of TCNB molecules along each direction (AB, AC and AD) are measured for simplification.

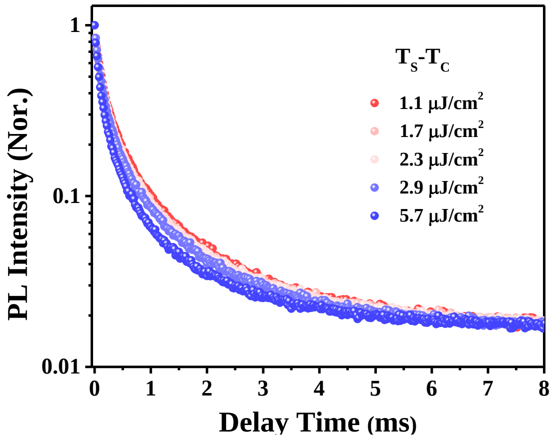

Supplementary Fig. 11 Sets of ms-scale PL kinetics of T_S-T_C under different 375 nm excitation intensities. When the excitation intensity is higher than 2.3 μJ/cm^2, the decay of PL kinetics becomes faster due to the occurrence of higher-order recombination.

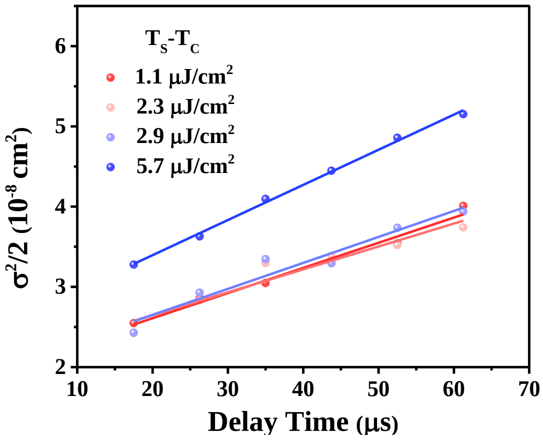

Supplementary Fig. 12 Diffusion coefficients obtained from the linear fitting of 1D time-dependent Gaussian variances ($\sigma^2(t)$) under different 375 nm excitation intensities. When the excitation intensity is higher than 2.9 μJ/cm^2, high order exciton recombination occurs, which leads to a positive deviation of the linear fit slope from those at low excitation intensity. In that case, the exciton density in areas closer to the excitation spot center is much higher than that at spot edge resulting in a faster PL decay due to the enhanced higher-order recombination. Therefore, the spatial nonuniform exciton decay will cause an artificial broadening of PL intensity distribution as delay time prolongs.

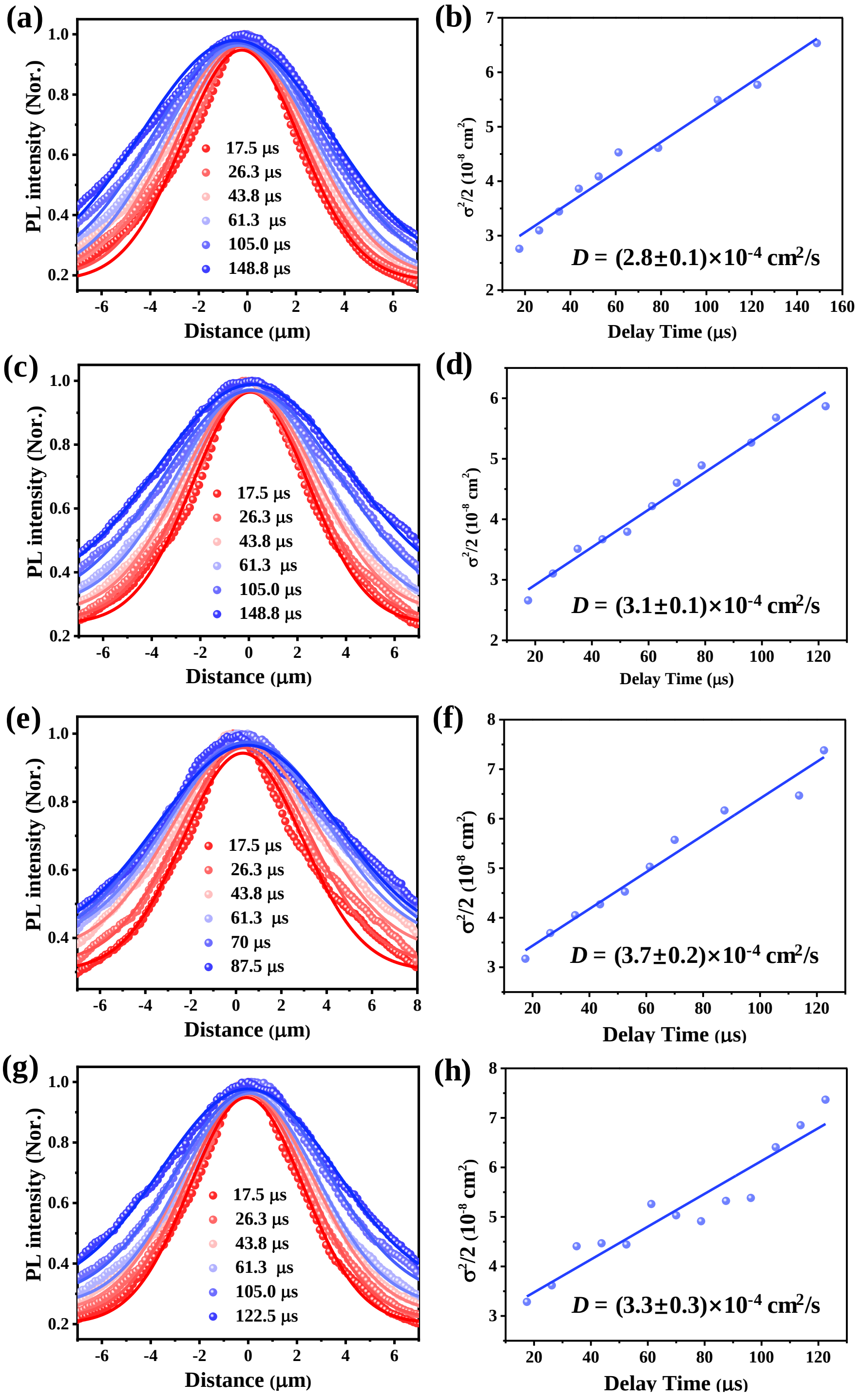

Supplementary Fig. 13 Millisecond-scale CT exciton transport in differtent T_S-T_C cocrystals. (a), (c), (e), (g) Normalized one-dimensional TADF intensity profiles of T_S-T_C fitted with Gaussian functions at different delay times. (b), (d), (f), (h) The determination of TADF-related exciton diffusion coefficient by linearly fitting the 1D time-dependent variances ($\sigma^2(t)$) of Gaussian profiles.

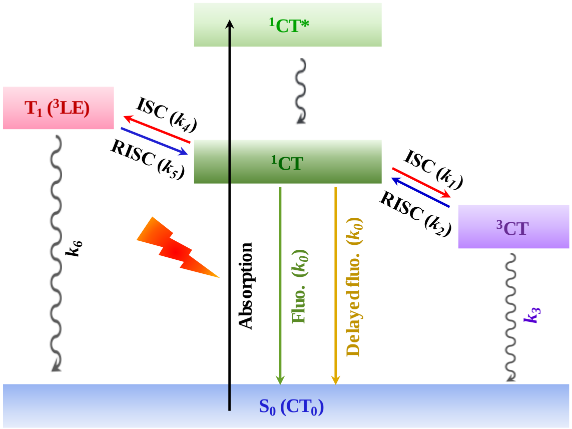

Supplementary Fig. 14 Diagram for all possible photophysical processes in T_S-T_C cocrystal where both T_1 and ^{3}CT are in dynamic equilibrium with ^{1}CT state.

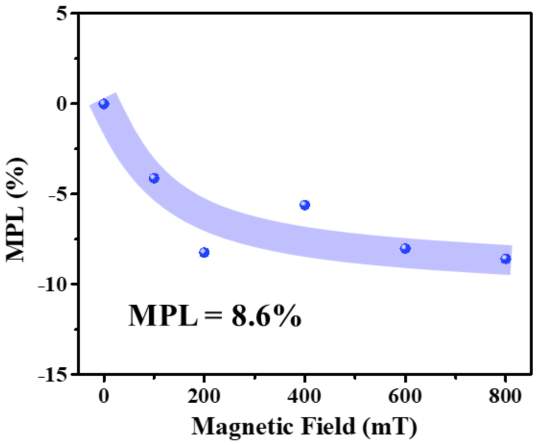

Supplementary Fig. 15 The magneto-photoluminescence (MPL) curve of T_S-T_C cocrystal up to the field strength of 800 mT. MPL signal can be observed when the ratio of singlet and triplet population is changed by an applied magnetic field, which is an indicator of triplet formation in organic semiconductors. MPL signal is given by $\mathrm{MPL} = \frac{\mathrm{PL_B} - \mathrm{PL_0}}{\mathrm{PL_0}} \times 100\%$, where PL_B and PL_0 represent PL intensity with and without applied magnetic field, respectively. The general MPL amplitude of intramolecular excited states (S_1 and T_1) is about 1%, while that of intermolecular CT excitons is about 5%. Herein, the obvious MPL effect with a value of ~8.6% provides direct evidence for the ^{3}CT dominated RISC in T_S-T_C.

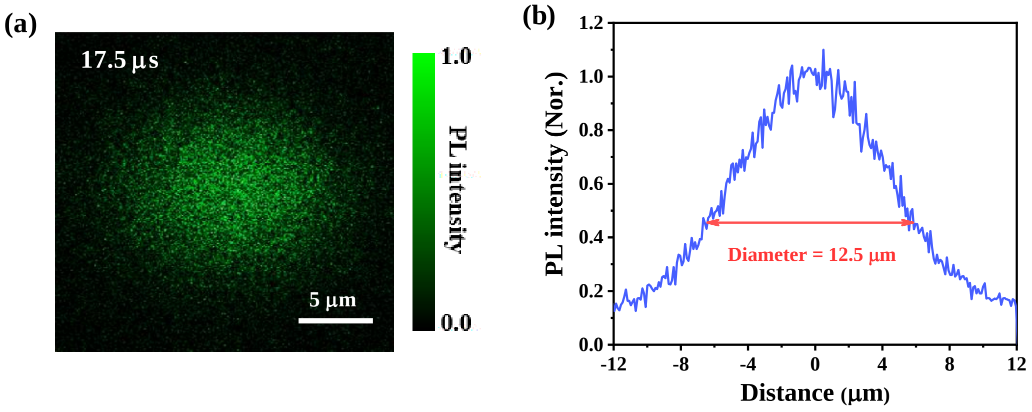

Supplementary Fig. 16 The initial TADF distribution at t = 17.5 μs after the defocused excitation. (a) TADF intensity image in T_S-T_C at t = 17.5 μs. The scanning image contains 256×256 pixels (98 nm/pixel), and the scale bar is 5 μm. (b) Normalized 1D PL intensity profile extracted from the *x*-axis in panel (a). The diameter of the initial TADF exciton distribution after the defocused excitation estimated to be ~12.5 μm.

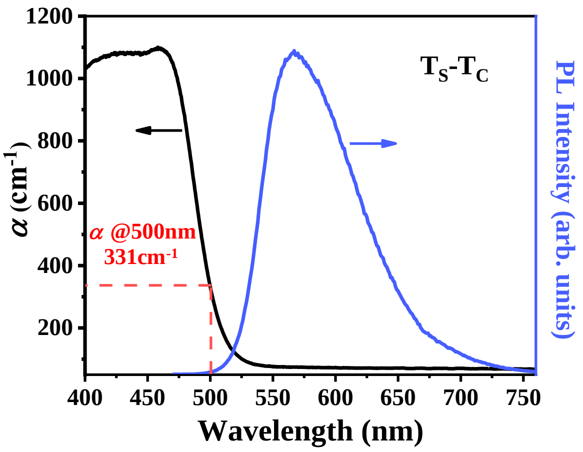

Supplementary Fig. 17 The absorption coefficient (α) and the PL spectra of T_S-T_C.

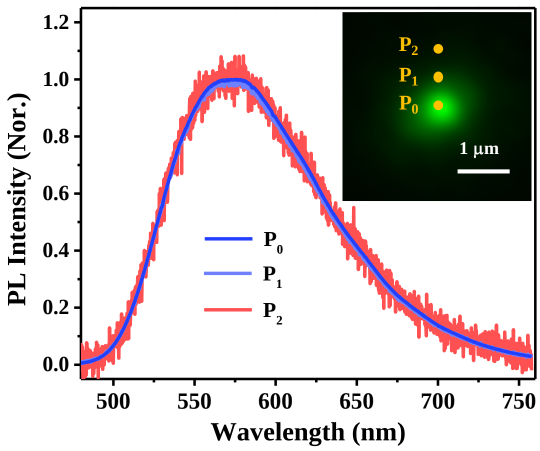

Supplementary Fig. 18 Normalized PL spectra of T_S-T_C collected at the excitation spot (P_0) and two selected sites (P_1 and P_2) with distances of 0.5 and 1.0 μm respectively. Insert is the TADF intensity image of T_S-T_C.

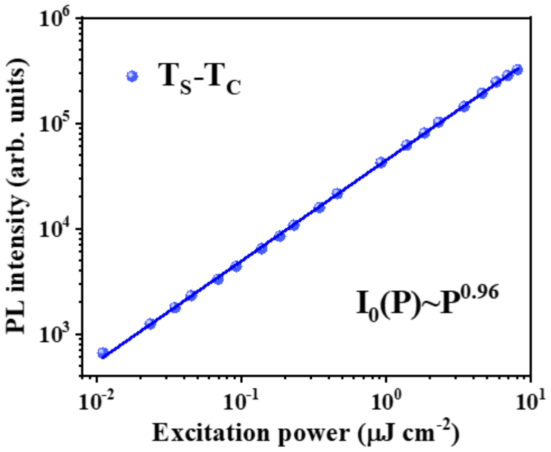

Supplementary Fig. 19 Initial maximum PL intensity (I_0, at delay time t ≈ 0 ps) as a function of excitation intensity (P) for T_S-T_C. The plot follows the law of $I_0 \propto P^{\alpha}$ (solid lines),where the α values is determined to be ~1, indicating that the major excited species are CT excitons in T_S-T_C.

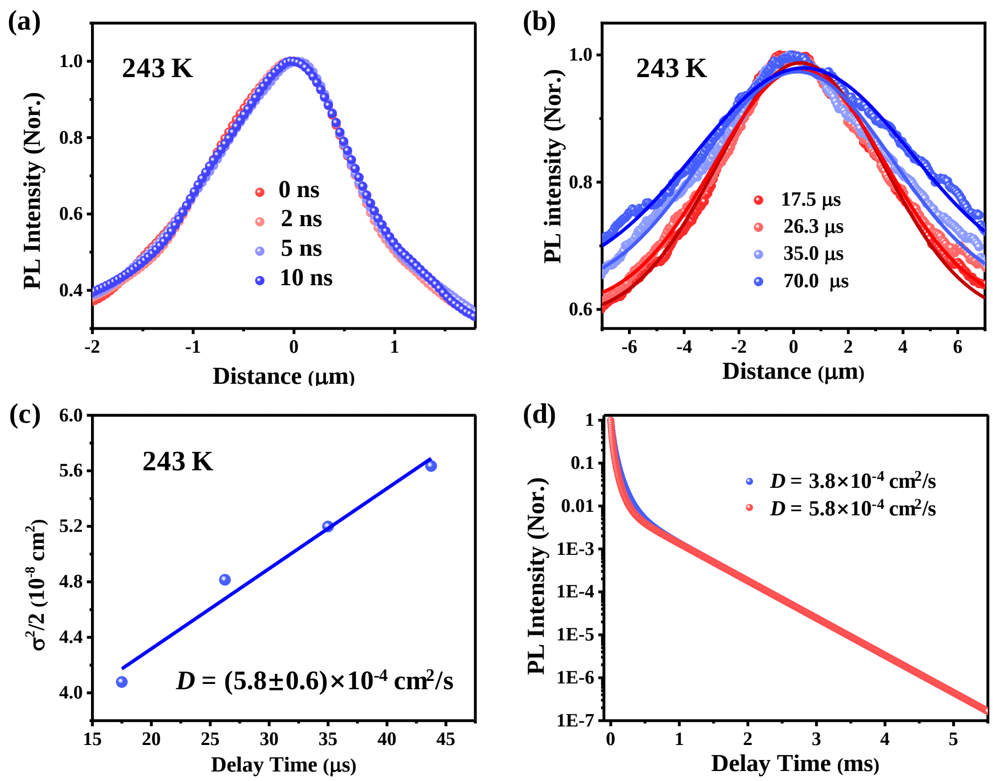

Supplementary Fig. 20 Imaging and modeling of CT exciton transport in T_S-T_C at 243 K. (a) Normalized 1D fluorescence intensity profiles at different delay times. The fluorescence distributions are almost unchanged, indicating a limited ^{1}CT transport distance within the spatial resolution (~58 nm) of ns-scale PL imaging measurements, which is similar to observations at RT shown in Fig. 2b. (b) Normalized 1D TADF intensity profiles at different delay times. Solid lines are the fitted curves by a Gaussian function. (c) Determination of TADF-related exciton diffusion coefficient at 243 K by the linearly fitting of 1D time-dependent Gaussian variances ($\sigma^2(t)$). The obtained D value of (5.8±0.6)×10^{-4} cm^2/s is slightly larger than that of (3.8±0.3)×10^{-4} cm^2/s at RT, providing direct evidence for the temperature-dependent Dexter-type ^{3}CT transport. There may be two possible reasons for the promoted Dexter-type CT transport at lower temperatures observed in T_S-T_C. First, the thermal motion of molecules is reduced as temperature drops, leading to a relatively fixed distance between adjacent molecules and thereby enhancing the overlap of their molecular orbitals. Second, the free energy of Dexter energy transfer may decrease due to the enhanced structural stability at lower temperatures. (d) Comparison of simulated TADF kinetics with different diffusion

coefficients (see Supplementary Note 3 for detailed simulation method). The influence of an increased D value on the fast component of TADF kinetics is quite small, which can hardly be identified in our measurements. Since the k_3 (the slow component of TADF kinetics) is also temperature-insensitive, the observed TADF kinetics are thus almost unchanged at different temperatures (Fig. 3b).

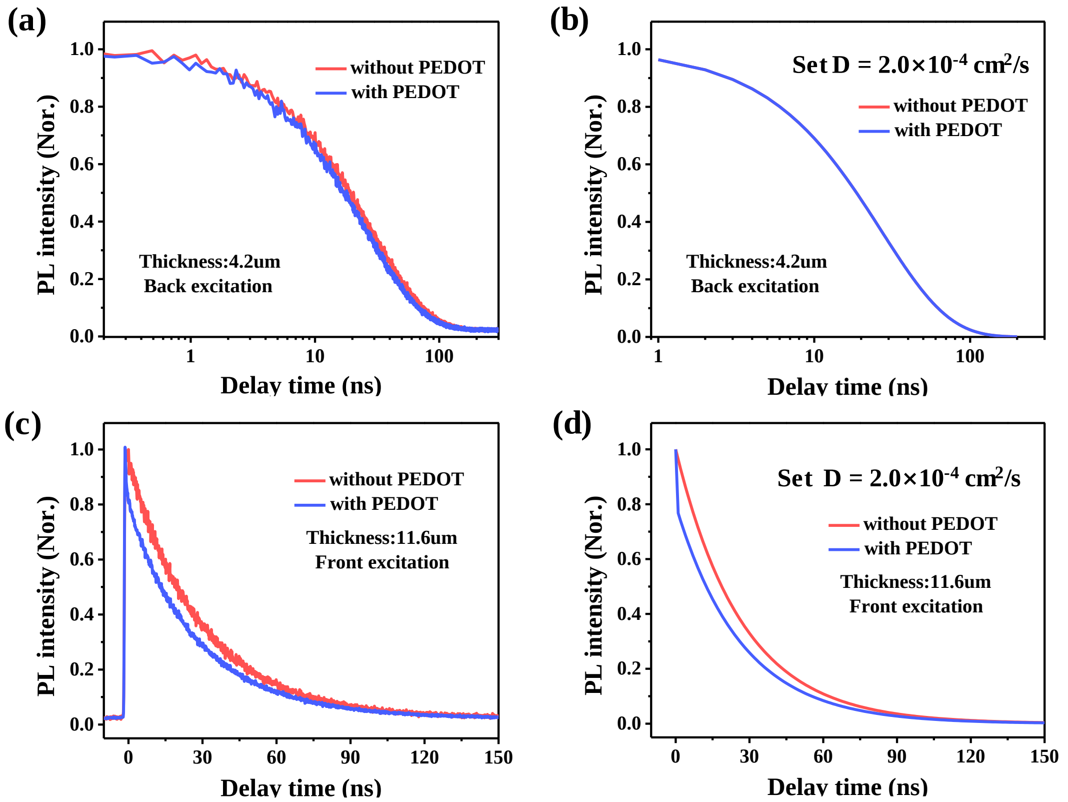

Supplementary Fig. 21 The estimation of ^{1}CT diffusion coefficient through PL quenching measurements. Experimental PL kinetics of T_S-T_C with and without applying PEDOT under the (a) back (T_S-T_C/substrate interface) and (c) front (T_S-T_C/PEDOT) interface excitation. The T_S-T_C cocrystal was synthesized on a glass substrate by the drop-casting method, and then PEDOT, functioned as the hole acceptor, was directly dropped on the top-surface of T_S-T_C. Simulations of PL kinetics of T_S-T_C with and without applying PEDOT under the (b) back (T_S-T_C/substrate interface) and (d) front (T_S-T_C/PEDOT) interface excitation based on 1D exciton diffusion and surface quenching model by setting the diffusion coefficient to 2.0×10^{-4} cm^2/s.

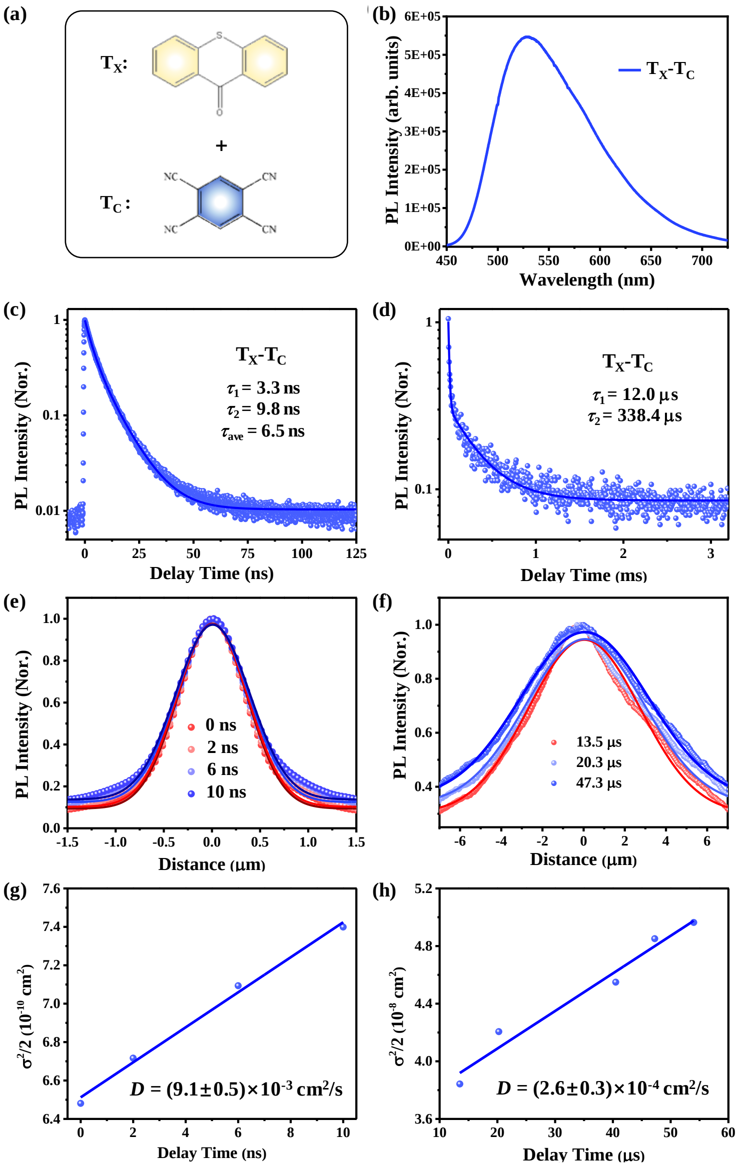

Supplementary Fig. 22 Imaging and modeling of CT exciton transport in T_X-T_C. (a) Chemical structures and (b) PL spectra of T_X-T_C cocrystal. PL kinetics of T_X-T_C collected on (c) nanosecond and (d) millisecond timescales. Solid lines in panels (c) and (d) are biexponential fittings of these kinetics. Normalized 1D (e) fluorescence and (f) TADF intensity profiles of T_X-T_C at different delay times, along with their Gaussian fittings. The determination of (g) ^{1}CT and (h) ^{3}CT diffusion coefficient by linearly fitting of the 1D time-dependent Gaussian variances ($\sigma^2(t)$). The ^{1}CT diffusion distance was calculated to be ~153.8 nm based on its average lifetime of 6.5 ns and diffusion coefficient of 9.1×10^{-3} cm^2/s. The ^{3}CT diffusion distance was calculated to be ~5.9 μm based on its intrinsic ^{3}CT lifetime of 338.4 μs and diffusion coefficient of 2.6×10^{-4} cm^2/s.

Supplementary Table 1. Crystallographic data for T_S-T_C measured at 301 K and 100 K.

**Compound**	**T_S-T_C at 301 K**	**T_S-T_C at 100 K**
Formula	C17 H8 N4	C34 H16 N8
Formula weight	268.27	536.55
Crystal system	Triclinic	Triclinic
Space group	*P -1*	*P -1*
Temperature, K	301	100
a, Å	7.2812(4)	7.1733(5)
b, Å	7.5794(6)	7.5366(5)
c, Å	12.7107(8)	12.5986(7)
α, deg	99.406(6)	99.959(5)
β, deg	93.036(5)	92.498(5)
γ, deg	94.522(5)	93.990(5)
Volume, $Å^3$	688.33(8)	668.15(8)
Z	2	1
Dc, g/ cm^3	1.294	1.333
μ, mm^{-1}	0.081	0.083
Reflections collected	7274	5134
2θ range for data collection, deg	3.256-63.93	3.288-67.52
F(000)	276.0	276.0
Goodness-of-fit on F^2	0.966	1.049
Final R indexes (I>2 (I))	R_1 = 0.0710, wR_2 = 0.1953	R_1 = 0.0633, wR_2 = 0.1519
Final R indexes (all data)	R_1 = 0.1221, wR_2 = 0.2215	R_1 = 0.1039, wR_2 = 0.1715

Supplementary Table 2. Fitting parameters of the delayed PL lifetimes shown in Fig.1d. The kinetics is fitted by a biexponential function ($y=\sum_i a_i \exp(-t/\tau_i)$).

**Cocrystal**	**τ_1 (μs) (a_1)**	**τ_2 (μs) (a_2)**
**T_S-T_C**	115.3 (71.3%)	818.4 (28.7%)

Supplementary References

1 Guo, Z., Manser, J. S., Wan, Y., Kamat, P. V. & Huang, L. Spatial and temporal imaging of long-range charge transport in perovskite thin films by ultrafast microscopy. *Nat. Commun.* **6**, 7471 (2015).
